# Spray‐Dried mRNA‐LNP Powder Enabling Multi‐Route Delivery With 3‐Year Refrigerated Stability

**DOI:** 10.1002/advs.78751

**Published:** 2026-11-10

**Authors:** Ashish Sarode, Priyal Patel, Frank DeRosa, Shrirang Karve, Regis Gervier

**Affiliations:** ^1^ mRNA Center of Excellence Waltham Massachusetts United States

**Keywords:** dry powder, lipid nanoparticles, mRNA therapeutics, mucosal, spray drying, thermostability

## Abstract

The clinical translation of mRNA‐lipid nanoparticle (mRNA‐LNP) therapeutics is constrained by limited stability of liquid formulations, necessitating ultra‐cold storage and restricting global accessibility. Here, we report a spray‐dried dry powder platform for mRNA‐LNPs that overcomes these limitations while enabling flexible routes of administration. Systematic evaluation of pharmaceutically relevant sugars identifies trehalose as an optimal matrix former, yielding high‐recovery mRNA‐LNP dry powder product (DPP) with controlled particle size, low residual moisture, and preserved LNP physicochemical properties upon reconstitution. Mechanistic studies combining solid‐state characterization, fluorescence tracking, and solvent extraction demonstrate that mRNA‐LNPs are entrapped within an amorphous trehalose matrix core, providing protection against dehydration and rehydration stress. Reconstituted DPP (RDPP) maintains in vivo protein expression comparable to liquid control (LC) following intravenous and intramuscular administration, while supporting effective mucosal delivery via pulmonary and sublingual routes. Direct administration of DPP without reconstitution enables lung‐targeted protein expression, highlighting potential for cold‐chain–independent therapies. Critically, DPP preserves colloidal stability, mRNA integrity, ionizable lipid stability, and biological activity for over 3 years under refrigerated conditions, whereas liquid formulation exhibits substantial degradation within 6–9 months. These findings establish spray‐dried mRNA‐LNP DPPs as a robust platform integrating long‐term stability with multi‐route delivery for improved accessibility of mRNA therapeutics.

## Graphical abstract

Spray‐dried mRNA‐LNP powder with trehalose matrix enables reconstitution for parenteral delivery or direct administration via pulmonary and sublingual routes, maintaining protein expression comparable to liquid controls. The thermostable powder preserves colloidal stability, mRNA integrity, and biological activity for over 3 years at 2°C–8°C, while liquid formulations degrade within 6–9 months, eliminating cold‐chain requirements.

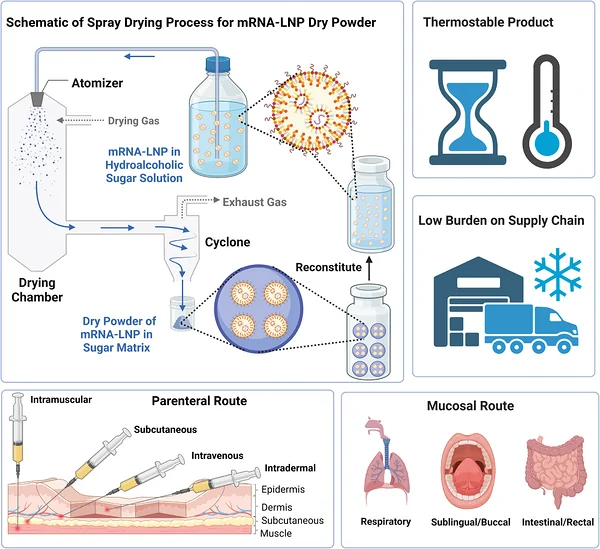

## 1 Introduction

mRNA has emerged as a transformative modality in modern medicine, offering unprecedented opportunities for disease prevention and treatment through programmable protein expression [1, 2]. The rapid development and clinical success of mRNA‐based COVID‐19 vaccines, including BNT162b2 (Comirnaty) and mRNA‐1273 (Spikevax), have validated the prophylactic vaccine potential of this platform technology and catalyzed global interest in expanding mRNA applications beyond infectious disease prevention to therapeutic applications [3, 4]. The versatility of mRNA technology positions it as a promising approach for protein replacement therapy, genetic disorders, gene editing applications, cancer immunotherapy, and regenerative medicine [5, 6, 7]. However, despite these remarkable advances, the clinical translation and global accessibility of mRNA therapeutics remain constrained by fundamental formulation challenges that limit their stability, distribution, and routes of administration [8, 9].

Current mRNA vaccines and therapeutics are predominantly formulated as LNP suspensions in liquid dosage forms, which require ultra‐cold storage conditions to maintain product stability and biological activity [10, 11]. The inherent chemical and physical instability of both mRNA molecules and lipid‐based delivery systems necessitates storage at temperatures ranging from −80°C to −20°C, creating substantial logistical barriers for global distribution, particularly in resource‐limited settings lacking robust cold‐chain infrastructure [12, 13]. These stringent storage requirements not only increase healthcare costs and limit vaccine and therapeutic accessibility but also restrict the practical application of mRNA therapeutics in point‐of‐care settings, emergency response scenarios, and regions with unreliable electricity supply [14]. Furthermore, the parenteral route of administration confines mRNA‐LNP products primarily to liquid dosage form presentation, typically requiring trained healthcare professionals for injection‐based delivery. This limitation reduces patient compliance, increases healthcare burden, and precludes exploration of other delivery routes using alternative dosage form presentation that could offer improved therapeutic outcomes, enhanced patient convenience, or tissue‐specific administration advantages [15].

The development of solid dosage forms represents a paradigm‐shifting approach to address these critical limitations and unlock the full therapeutic potential of mRNA technology. Solid formulations inherently offer superior thermostability compared to their liquid counterparts by eliminating water‐mediated degradation pathways, reducing molecular mobility, and providing a protective matrix that shields both mRNA and lipid components from environmental stressors [16, 17]. This enhanced stability profile could enable storage at standard refrigeration temperatures (2°C–8°C) or even ambient conditions, dramatically simplifying supply chain logistics and expanding global accessibility. Beyond stability advantages, solid dosage forms provide unprecedented flexibility in dosage form presentation and administration routes. Dry powder formulations can be engineered into diverse formats including inhalable powders for pulmonary delivery, sublingual or buccal powders/tablets/films for mucosal absorption, orally disintegrating films for pediatric or geriatric populations, transdermal patches for sustained release, and implantable depots for long‐term protein expression [18]. Each of these alternative delivery routes offers distinct therapeutic advantages – pulmonary administration enables direct targeting of respiratory tissues for infectious diseases or lung cancer [19]; sublingual delivery bypasses first‐pass metabolism and offers rapid systemic absorption [20]; oral formulations maximize patient compliance and enable self‐administration [21]; and transdermal systems provide controlled, sustained protein expression for chronic conditions [22].

Among various solidification technologies available in pharmaceutical manufacturing, spray drying emerges as a particularly attractive approach for mRNA‐LNP formulation development. Spray drying is a well‐established, scalable, and economically viable unit operation that has been successfully employed for decades to manufacture dry powder formulations of small molecules, peptides, proteins, and vaccines [23, 24]. The process involves atomization of a liquid feed into fine droplets followed by rapid solvent evaporation in a controlled temperature environment, yielding uniform particulate powders with tailored physicochemical properties. The continuous nature of spray drying operations, combined with precise control over process parameters such as inlet temperature, feed rate, atomization pressure, and drying gas flow, enables reproducible manufacturing of powders with defined particle size distribution, moisture content, bulk density, and flowability characteristics [25]. Critically, the rapid evaporation kinetics inherent to spray drying minimize thermal exposure time, making the process suitable for thermolabile biologics including nucleic acids and lipid‐based delivery systems [26]. The resulting spray‐dried powders can be further processed using conventional pharmaceutical equipment such as blenders, granulators, roller compactors, capsule fillers, and tablet presses to generate finished dosage forms optimized for specific administration routes and patient populations.

Despite the compelling rationale and established manufacturing infrastructure for solid dosage forms, the application of spray drying technology to mRNA‐LNP systems remains largely unexplored. The complex supramolecular architecture of LNPs, comprising ionizable lipids, phospholipids, cholesterol, and polyethylene glycol‐lipid conjugates assembled around negatively charged mRNA cargo, presents unique formulation challenges during dehydration/rehydration processes [10, 27]. The removal of water from LNP suspensions by spray drying to generate DPP, followed by reconstitution either before or after administration, must preserve the structural integrity of lipid bilayers, maintain mRNA encapsulation efficiency, prevent particle aggregation or fusion, and retain the biological activity necessary for cellular uptake and protein translation [28]. Previous attempts to solidify mRNA‐LNP formulations through lyophilization (freeze‐drying) have yielded mixed results, with concerns regarding process economics, batch‐to‐batch variability, particle uniformity in the reconstituted state, and limited flexibility for downstream processing into alternative dosage form presentations [29]. The batch nature of lyophilization, combined with extended processing times often exceeding 48–72 h, further limits its scalability for commercial manufacturing of mRNA therapeutics.

While previous work demonstrated the feasibility of spray‐dried mRNA‐LNP dry powders for inhalation‐based pulmonary delivery [30, 31, 32], the current study pivots toward a ready‐to‐reconstitute powder dosage form presentation that broadens the therapeutic reach of mRNA‐LNP technology across mucosal as well as parenteral administration routes without compromising biological activity or colloidal stability. We hypothesize that rational selection of solvents and stabilizing excipients, combined with optimized spray drying parameters, can yield solid dosage forms of highly potent mRNA‐LNPs that maintain colloidal stability and structural integrity, preserve mRNA stability, and retain biological activity comparable to liquid formulations. By establishing spray drying as a viable manufacturing platform for mRNA therapeutics, this work aims to expand the clinical applications of mRNA technology beyond current limitations and enhance global accessibility of this transformative therapeutic modality.

## 2 Results and Discussion

### 2.1 Strategic Development and Comprehensive Evaluation of Thermostable mRNA‐LNP DPP Technology

The development and evaluation of ready‐to‐reconstitute mRNA‐LNP DPP followed a systematic and integrated workflow (Figure 1) designed to establish a robust platform for thermostable mRNA therapeutics. This comprehensive approach encompassed multiple critical stages: (i) formulation optimization through systematic screening of pharmaceutically relevant sugars to identify optimal stabilizing excipients, (ii) spray drying process development with both open and closed loop configurations to establish scalable manufacturing parameters (Figure 1a), (iii) comprehensive physicochemical characterization of DPP morphology, particle architecture, and solid‐state properties to elucidate stabilization mechanisms, (iv) in‐vivo efficacy assessment in murine models via parenteral (Figure 1b) and mucosal (Figure 1c) routes to validate preserved biological activity across diverse administration pathways, and (v) long‐term stability evaluation under pharmacy‐friendly refrigerated storage conditions (2°C–8°C) for up to 3.5 years to demonstrate superior thermostability compared to conventional liquid formulations (Figure 1d).

**FIGURE 1:**
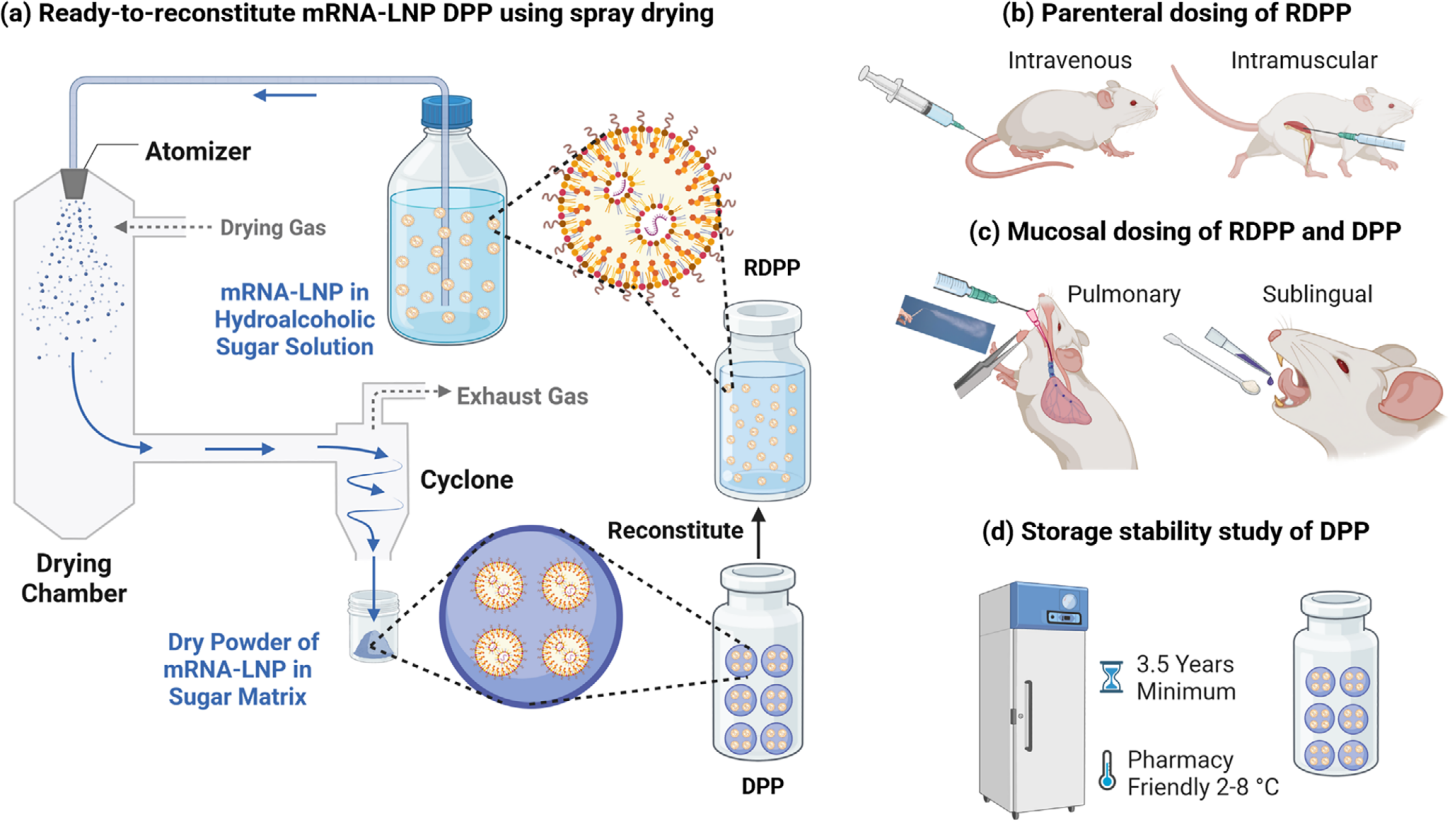
Schematic overview of mRNA‐LNP DPP development, characterization, and evaluation workflow. (a) Spray drying manufacturing process of hydroalcoholic mRNA‐LNP suspension with sugar excipients to produce ready‐to‐reconstitute DPP with mRNA‐LNPs entrapped in the core of an amorphous sugar matrix. (b) Parenteral administration route evaluation using RDPP via intravenous and intramuscular injection. (c) Mucosal administration route assessment using both RDPP and DPP administered as‐is via pulmonary and sublingual routes. (d) Long‐term storage stability evaluation of DPP under pharmacy‐friendly refrigerated conditions (2°C–8°C) for up to 3.5 years.

### 2.2 Sugar Excipient Optimization Reveals Trehalose as Superior Stabilizer for Spray‐dried mRNA‐LNP Formulations

As described in previous invention by Sarode et al., a panel of pharmaceutically relevant sugars including lactose, xylitol, mannitol, sucrose, and trehalose was evaluated for their ability to facilitate spray drying of mRNA‐LNPs [33]. Spray drying of hydroalcoholic mRNA‐LNPs formulated with ionizable lipid cKK‐E10 containing these sugars at their post‐reconstitution concentrations of 1.25%–7% w/v revealed substantial differences among sugars in both DPP manufacturability and RDPP quality attributes (Figure 2). Lactose and xylitol did not yield recoverable powders at any concentration. The majority of the material adhered to the cyclone walls, consistent with their low glass transition temperature (Tg) and high hygroscopicity, which promote particle stickiness and insufficient solidification during spray drying. This behavior aligns with the broader understanding that low Tg saccharides readily enter a rubbery state under typical drying temperatures, leading to wall deposition and reduced yield, a phenomenon well described for amorphous excipients in spray‐dried protein and biologic matrices [34].

**FIGURE 2:**
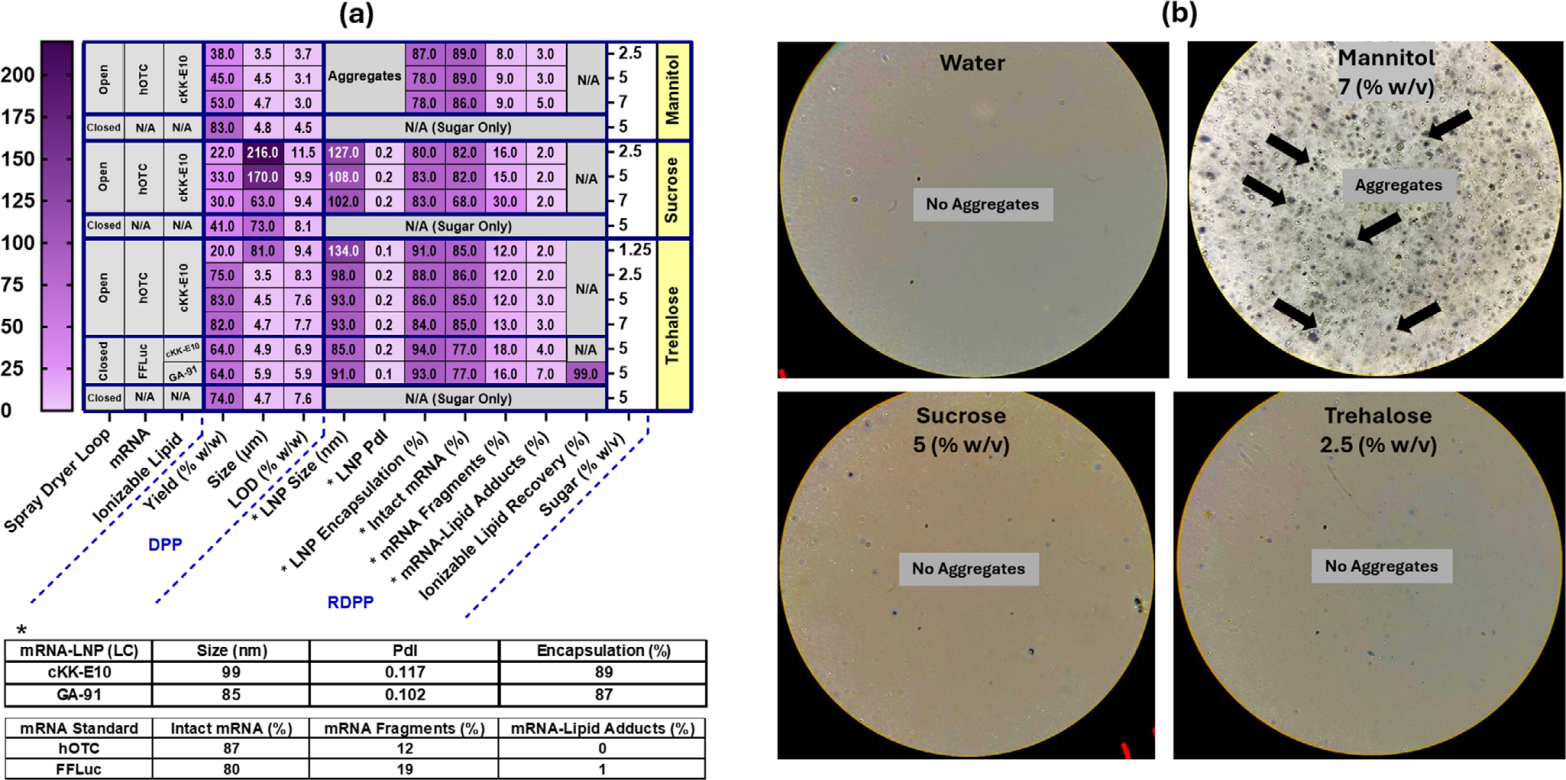
Impact of sugar type and concentration on DPP and RDPP quality attributes. (a) Heat map summarizing the effect of mannitol, sucrose, and trehalose at post‐reconstitution concentration of 1.25–7% w/v on DPP and RDPP quality attributes. Mannitol yielded high recovery but caused LNP aggregation upon reconstitution. Trehalose demonstrated superior performance with the highest yields (75%–82%), optimal particle sizes (3–5 µm), low moisture content, and excellent LNP preservation. Closed loop spray drying showed comparable quality to open loop. The reported values represent mean ± 10% SD (*n* = 3). (b) Light microscopic images (400×) of RDPPs illustrating sugar‐dependent reconstitution behavior, including LNP aggregation with mannitol and uniform dispersion with sucrose and trehalose formulations.

In contrast, mannitol readily formed dry powders across concentrations, showing steadily increasing yields and desirable particle sizes in the 3–5 µm range, with loss on drying (LOD) values below 4% w/w (Figure 2a). These observations reflect intrinsically crystalline nature and high melting point of mannitol, which facilitate rapid particle solidification and reduce droplet adhesion to dryer surfaces. The superior performance of mannitol is consistent with its role as a crystallizing scaffolding agent described in spray‐dried protein formulations, where its crystalline domains help preserve particle morphology but fail to produce a protective amorphous matrix around biomolecules [35]. Correspondingly, RDPPs containing mannitol exhibited substantial LNP aggregation upon reconstitution at all concentrations (Figure 2b). Crystallization of mannitol during drying limits its ability to immobilize lipid structures and restrict fusion events, leading to poor preservation of nanoparticle architecture despite retention of mRNA integrity. Similar limitations of sugar‐alcohol crystallization in stabilizing lipid‐based carriers have been noted in spray‐dried nanoparticle systems formulated with trehalose‐dominant matrices, where the amorphous component provides the essential protective effect [36].

Sucrose produced DPPs with moderate yields (22–33% w/w), lower than mannitol but sufficient for characterization (Figure 2a). Powder particles were larger at lower sucrose concentrations, consistent with decreased solids content affecting droplet‐to‐particle conversion efficiency. The relatively high LOD values, particularly at low sucrose levels, reflect the hygroscopic behavior and moderate Tg of sucrose, which increase moisture uptake and reduce powder stability. These characteristics are consistent with literature describing limited ability of sucrose to maintain a stable glassy matrix at elevated relative humidity compared with trehalose [37]. Despite these processing limitations, sucrose effectively prevented LNP aggregation upon reconstitution (Figure 2b). The widely documented ability of sucrose to form hydrogen‐bonded amorphous matrices, inhibit lipid fusion, and protect biologics during dehydration makes it a well‐established lyoprotectant and spray‐drying excipient [38]. RDPPs displayed acceptable physicochemical attributes, including size, PdI, and encapsulation efficiency. At higher sucrose concentrations, however, a modest rise in mRNA fragmentation was observed (Figure 2a). This may reflect sucrose's relatively higher moisture retention and potential to promote hydrolytic degradation, consistent with reports of comparative instability of sucrose against acid‐catalyzed reactions and its lower resistance to hydrolysis relative to trehalose [39].

Trehalose consistently yielded the most favorable outcomes. DPPs containing trehalose exhibited the highest yields (75–82% w/w at 2.5–7% w/v), stable particle sizes between 3 and 5 µm, and lower LOD values than sucrose DPPs (Figure 2a). The superior performance of trehalose corresponds with its high Tg, relatively stable glass matrix, strong water‐replacement capability, and exceptional capacity to preserve lipid bilayer spacing and biomolecular structure during dehydration. These properties are extensively documented in both protein stabilization and microencapsulation literature, where trehalose maintains its glassy state over a broader humidity range relative to sucrose, conferring greater physical stability to dried matrices [40, 41, 42, 43]. Upon reconstitution, trehalose formulations exhibited no visible aggregation (Figure 2b), and RDPPs maintained smaller LNP sizes and higher encapsulation efficiencies compared with both mannitol and sucrose (Figure 2a). mRNA integrity was preserved across all tested concentrations. Although trehalose at 1.25% w/v also supported spray drying, the reduced solids content resulted in diminished yield and larger particle and LNP sizes in both DPP and RDPP, respectively, consistent with the general requirement for sufficient matrix‐forming excipient to achieve optimal particle morphology [44].

Across all sugars tested, the results underscore the importance of excipient physicochemical properties in determining both spray‐drying feasibility and LNP preservation. Crystalline sugars such as mannitol promote powder formation but lack the amorphous matrix necessary to stabilize LNPs through dehydration–rehydration stress, while glass‐forming disaccharides provide superior structural stabilization at both the particle and molecular levels. Trehalose, in particular, exhibits a combination of high‐Tg matrix formation, chemical stability, and lipid‐interfacing protection that collectively enhanced DPP recovery and RDPP quality. Based on these advantages, trehalose at a post‐reconstitution concentration of 5% w/v was selected as the lead formulation for further development. While the LOD method was used to estimate relative moisture content during the initial screening of various sugars, the moisture content of the lead DPP formulations selected for in vivo expression testing and thermostability studies was measured more accurately using the Karl Fisher (KF) titration. The 5% w/v trehalose‑based lead DPPs demonstrated acceptable moisture contents of 3–4% w/w using KF that previously showed an LOD value of 7.7% w/w during initial screening (Figure 2a), consistent with values commonly observed for spray‑dried DPPs [23, 45, 46].

The feasibility of using closed (inert) loop to manufacture mRNA‐LNP DPP using spray drying was evaluated for potential successful technology transfer and scale up. The transition from open to closed loop systems alters process dynamics and affects heat and mass transfer characteristics, potentially impacting critical quality attributes such as LNP integrity, mRNA encapsulation efficiency, and particle morphology [23]. Establishing comparability requires systematic evaluation of particle size distribution, residual moisture, reconstitution properties, mRNA stability, and lipid degradation profiles across both configurations. Early feasibility confirmation prevents costly late‐stage delays and ensures that the operational advantages of closed loop such as enhanced containment, reduced risk of nuclease exposure, and integrated cleaning capabilities do not compromise product characteristics established during initial development, supporting robust scale‐up to commercial manufacturing. As shown in the heat map (Figure 2a), all quality attributes of the lead DPP and its RDPP formulated using FFLuc mRNA and two distinct ionizable lipids (cKK‐E10 and GA‐91) at a post reconstitution trehalose concentration of 5% (w/v) remained comparable to the open loop product at the corresponding trehalose concentration.

### 2.3 Core Entrapment Architecture and Solid‐State Characterization Define DPP Stabilization Mechanisms

The scanning electron microscopy (SEM) images of the mRNA‐LNP DPPs manufactured using various sugars at a post‐reconstitution sugar concentration of 5% (w/v) are shown in Figure 3. The SEM images of mRNA‐LNP DPPs of other sugar concentrations and those of DPPs of pure sugars are shown in Figure S2. All the DPPs were spherical in shape with relatively smooth surfaces, with their individual particle sizes matching the data obtained using Mastersizer (Figure 2a). For example, the sucrose‐based product showed larger particles of approximately 150–200 µm in size (Figure 3b), whereas the individual particles of mRNA‐LNP DPPs of both mannitol (Figure 3a) and trehalose (Figure 3c) appeared below 5 µm. To determine whether mRNA‐LNPs were entrapped within the precipitated sugar matrix core or merely coating the surface of the DPP, rhodamine‐DOPE ((1,2‐dioleoyl‐sn‐glycero‐3‐phosphoethanolamine‐N‐(lissamine rhodamine B sulfonyl) (ammonium salt)) labeled mRNA‐LNP‐based DPP was manufactured and subjected to two distinct ethanol treatments – vigorous vortexing or overnight stirring with a detailed description shown in Table S1. The supernatant was collected and the resultant DPP was dried in a vacuum desiccator. SEM imaging of the dried DPP demonstrated that vigorous ethanol vortexing preserved DPP morphology and surface characteristics without significant alteration as compared to that of the untreated DPP (Figure 3d,e). In contrast, overnight ethanol stirring caused observable deformation of the spherical structure with apparent particle shrinkage, indicating partial LNP product loss through gradual ethanol permeation into the DPP matrix (Figure 3f).

**FIGURE 3:**
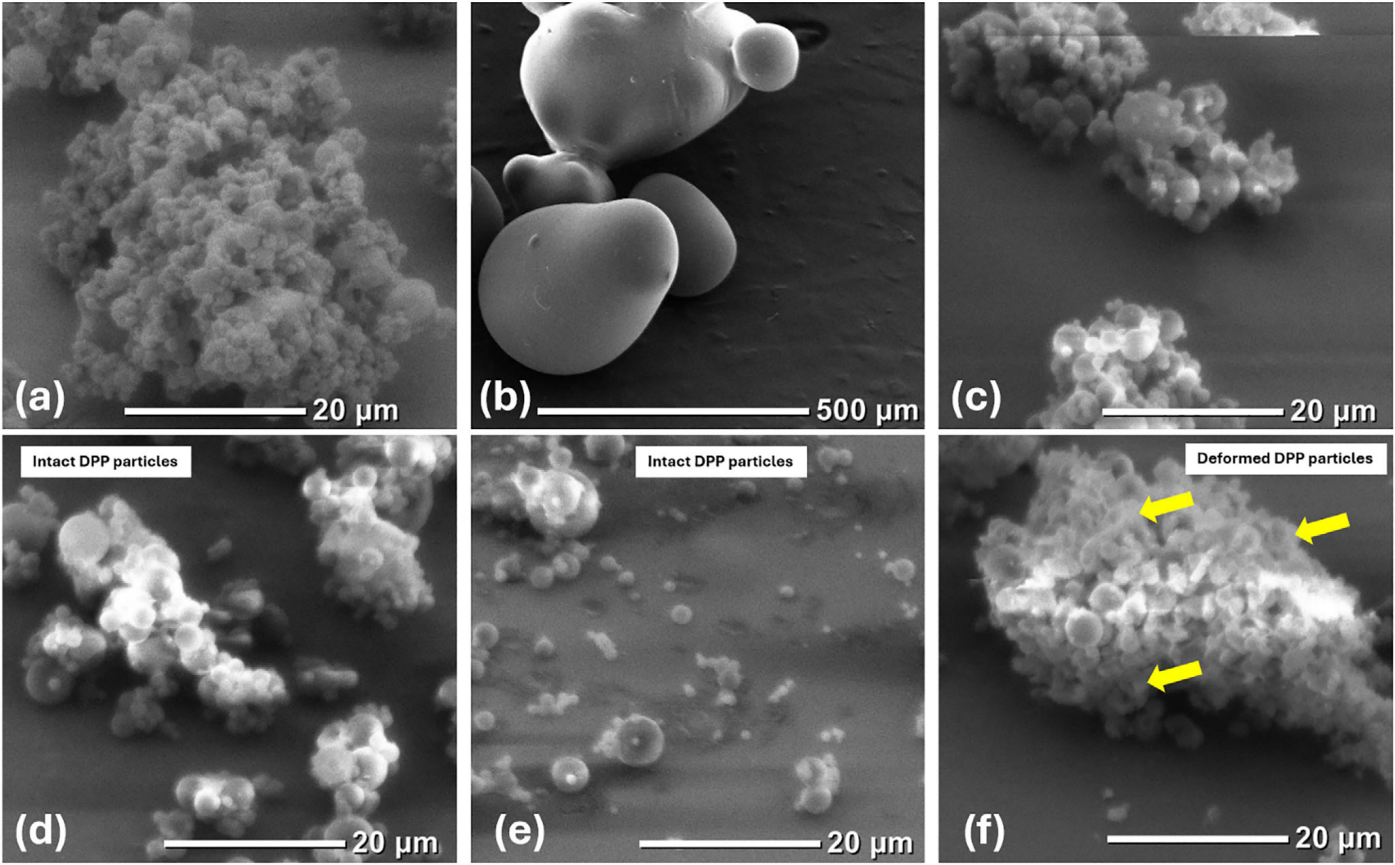
SEM analysis of mRNA‐LNP DPP morphology and matrix integrity. SEM images of mRNA‐LNP DPPs formulated at 5% w/v post‐reconstitution sugar concentration with (a) mannitol, (b) sucrose, and (c) trehalose, showing spherical particles with smooth surfaces and sizes consistent with laser diffraction data. Ethanol treatment of rhodamine‐DOPE‐labeled DPPs at 5% w/v post‐reconstitution trehalose concentration: (d) untreated control, (e) after vigorous vortexing (preserved morphology), and (f) after overnight stirring (particle shrinkage and deformation), demonstrating mRNA‐LNP entrapment within the sugar matrix core rather than surface coating.

These morphological observations were further validated through multiple visual and quantitative analytical approaches (Figure 4). The DPP with overnight ethanol washing showed visible loss of pink color, whereas both untreated and vigorously vortexed samples appeared similar in pink shade, providing visual confirmation of LNP extraction (Figure 4a). Fluorescence measurements of the supernatant showed higher levels for overnight ethanol treatment compared to vigorous vortexing, directly confirming greater LNP release (Figure 4a,b). Correspondingly, overnight stirring resulted in decreased fluorescence, increased LNP size and PdI, decreased mRNA recovery, and visible aggregate formation in the RDPP (Figure 4a,b). Collectively, these comprehensive SEM and characterization data provide definitive evidence that mRNA‐LNPs are entrapped within the sugar matrix core of the DPP rather than surface‐adsorbed. This core entrapment mechanism represents significant advancement, since it is expected to fundamentally enhance both the colloidal and chemical stability of the mRNA‐LNP system during storage and handling, thereby addressing critical stability challenges in mRNA vaccines and therapeutics development.

**FIGURE 4:**
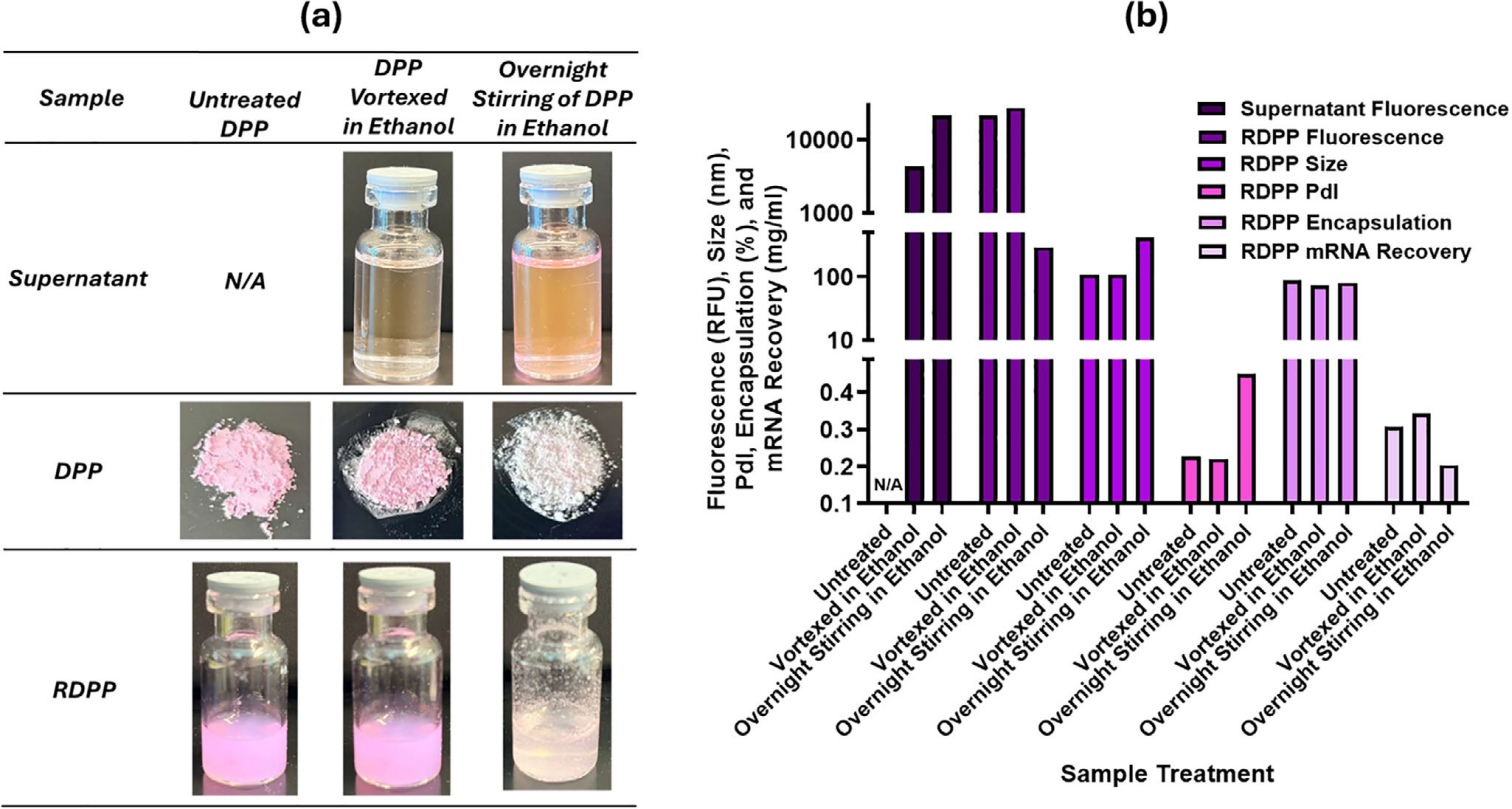
Visual and quantitative validation of mRNA‐LNP entrapment within DPP matrix. (a) Photographs of rhodamine‐DOPE‐labeled DPPs at 5% w/v post‐reconstitution trehalose concentration showing color retention in untreated and vigorously vortexed samples versus visible pink color loss after overnight ethanol stirring, with corresponding supernatant exhibiting increased pink fluorescence visibly and RDPP photographs visibly demonstrating decreased fluorescence and aggregate formation upon partial LNP extraction. (b) Quantitative analysis of ethanol supernatant fluorescence, RDPP particle size, PdI, encapsulation, and mRNA recovery, confirming greater LNP release with overnight stirring compared to vigorous vortexing. Data collectively demonstrate core entrapment of mRNA‐LNPs within the sugar matrix rather than surface adsorption.

Powder X‐ray diffraction (PXRD) (Figure 5a) and modulated differential scanning calorimetry (MDSC) (Figure 5b–d) of spray‐dried mannitol, trehalose, sucrose, and their corresponding mRNA‐LNP DPPs formulated at a post‐reconstitution sugar concentration of 5% (w/v) revealed distinct solid‐state behavior and process performance. The PXRD profiles of mRNA‐LNP DPPs at other sugar concentrations and pure sugar DPPs are shown in Figure S3, while the corresponding MDSC profiles are shown in Figure S4. Mannitol exhibited sharp diffraction peaks in PXRD, suggesting a predominantly crystalline phase, and this crystalline character persisted in the corresponding mRNA‐LNP DPP (Figure 5a). In the first heating cycle of MDSC, mannitol showed a sharp melting endotherm at ∼165°C, with the corresponding mRNA‐LNP DPP displaying similar melting behavior at ∼163°C (Figure 5b). During the cooling cycle, mannitol showed a pronounced crystallization exotherm at ∼120°C, which could indicate melt–recrystallization behavior and a strong intrinsic tendency to crystallize that persisted even in the corresponding mRNA‐LNP DPP (Figure 5c). Similarly, in the second heating cycle, mannitol as well as its mRNA‐LNP DPP retained the melting transition at ∼163‐165°C, suggesting stable crystallinity in both mannitol‐only and mRNA‐LNP DPPs (Figure 5d).

**FIGURE 5:**
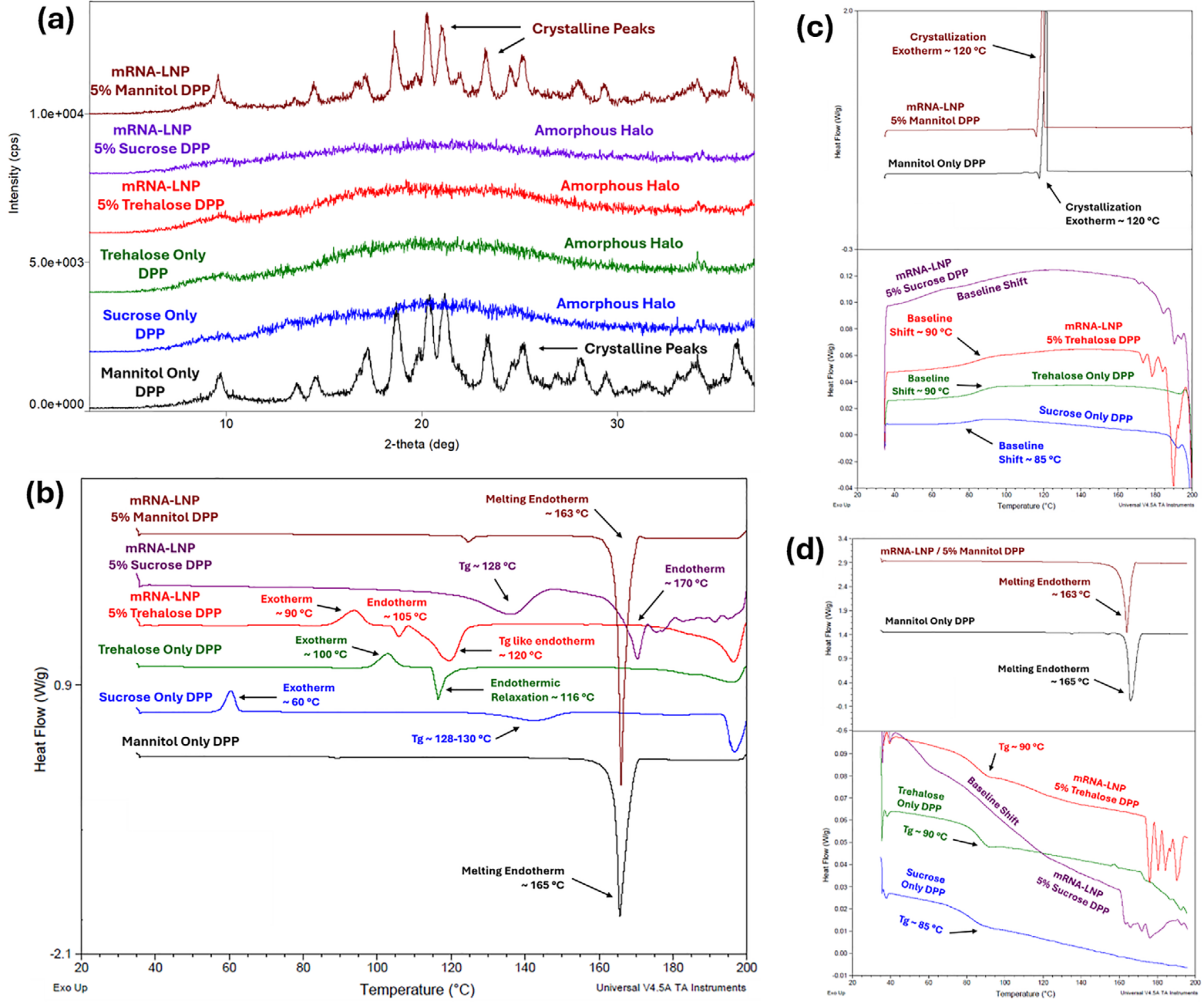
Solid‐state characterization of sugar‐only and mRNA‐LNP DPPs at 5% w/v post‐reconstitution concentration. (a) PXRD patterns showing crystalline peaks for mannitol and amorphous halos for trehalose and sucrose. MDSC thermograms: (b) first heating cycle revealing mannitol melting (∼165°C), trehalose relaxation events (∼100°C–120°C), and sucrose moisture‐driven transitions (∼60°C–130°C); (c) cooling cycle showing glass formation in trehalose (∼90°C) and sucrose (∼85°C), and mannitol crystallization (∼120°C); (d) second heating cycle demonstrating equilibrated glass transitions for trehalose and sucrose and persistent crystallinity in mannitol‐based formulations.

Trehalose exhibited amorphous halos in PXRD, suggesting vitrification, and retained similar solid‐state behavior in the corresponding mRNA‐LNP DPP (Figure 5a). In the first heating cycle, trehalose displayed an exotherm at ∼100°C followed by a small endothermic relaxation at ∼116°C (Figure 5b). The corresponding mRNA‐LNP DPP showed similar first‐cycle behavior – an exotherm at ∼90°C, a smaller endotherm at ∼105°C, and a Tg‐like endotherm at ∼120°C, which could be consistent with sequential structural relaxation of a highly non‐equilibrated amorphous matrix in both trehalose‐only as well as the corresponding mRNA‐LNP DPPs. During the cooling cycle, both trehalose and its mRNA‐LNP DPP exhibited a baseline shift at ∼90°C, indicative of glass formation (Figure 5c). In the second heating cycle, both products converged to a single Tg value (∼90°C), suggesting irreversible equilibration of the amorphous system (Figure 5d).

Sucrose exhibited amorphous halos by PXRD (Figure 5a), suggesting vitrification, but showed signs of altered amorphous structure in the corresponding mRNA‐LNP DPP with possible lipid–sucrose interactions. In the first heating cycle, sucrose showed an exothermic event near ∼60°C and an apparent glass transition around ∼128°C–130°C, which could reflect moisture‐driven heterogeneity and evolving glass structure (Figure 5b). The corresponding mRNA‐LNP DPP of sucrose showed disappearance of the first‐cycle relaxation and a Tg near ∼128°C followed by a higher‐temperature endotherm (∼170°C) due to possible lipid–sucrose interaction (Figure 5b). During the cooling cycle, sucrose exhibited a baseline shift at ∼85°C (Figure 5c), indicative of glass formation that converged to a single Tg value (∼85°C) in the second heating cycle (Figure 5d). On the other hand, the corresponding mRNA‐LNP DPP showed a broad baseline shift in both cooling and second heating cycles, further supporting altered amorphous structure (Figure 5c,d). Sucrose‐based mRNA‐LNP DPP also produced larger particle sizes and lower yield compared to the corresponding trehalose‐ and mannitol‐based mRNA‐LNP DPPs (Figure 2a), which could be consistent with increased stickiness and particle coalescence due to excessive moisture induced plasticity.

These differences likely evolved from fundamental material properties governing crystallization tendency, hygroscopicity, and glass stability. Mannitol, with relatively high molecular symmetry and low configurational entropy in the melt, promoted rapid nucleation and crystal growth during drying and cooling, leading to a stable crystalline phase and high process yield. Trehalose, in contrast, acted as a strong glass former with a high intrinsic Tg and resistance to crystallization, attributable to its hydrogen‐bonding network and reduced molecular mobility in the supercooled state. Rapid spray drying at low outlet temperature produced a highly non‐equilibrated glass with excess free volume and elevated fictive temperature, resulting in sequential relaxation events (exotherm followed by endotherm) during first heating. Sucrose exhibited intermediate behavior but higher hygroscopicity and susceptibility to moisture‐induced plasticization, leading to heterogeneous amorphous domains with locally depressed Tg, early‐stage relaxation (∼60°C), and a dynamically shifting apparent Tg during heating. The larger particle size and lower yield of sucrose formulations were attributed to surface plasticization, increasing interparticle cohesion and wall deposition.

These solid‐state characteristics have important implications for storage stability of mRNA‐LNP DPPs. In the presence of lipids from mRNA‐LNPs, trehalose maintained a relatively stable amorphous matrix with minimal changes in Tg, suggesting limited interaction and preservation of glass rigidity. Sucrose, however, showed suppression of relaxation events and broadened thermal transitions, which suggested that lipid incorporation reduced moisture heterogeneity but introduced a more plasticized and less well‐defined amorphous structure. At 2°C–8°C, both systems would be below their bulk Tg; however, trehalose could provide a more homogeneous, high‐Tg matrix that may effectively immobilize the lipid phase, minimizing molecular mobility, phase separation, and aggregation. In contrast, sucrose could remain more susceptible to moisture‐induced local plasticization, leading to regions of higher mobility that might promote lipid reorganization, fusion, or leakage over time. Overall, these solid‐state characteristics suggest that while mannitol favored crystallization with high yield, trehalose may provide a more robust amorphous matrix and better balance of processability and stability, whereas sucrose may form a more heterogeneous and potentially less stable amorphous system during long‐term storage.

### 2.4 Preserved Therapeutic Efficacy Across Multiple Administration Routes Validates DPP Clinical Translation Potential

Lead DPPs were prepared at a post‐reconstitution trehalose concentration of 5% (w/v) and formulated with human ornithine transcarbamylase (hOTC), human erythropoietin (hEPO), and firefly luciferase (FFLuc) mRNAs to assess in vivo protein expression. Two chemically distinct ionizable lipid based LNP systems, cKK‐E10 and GA‐91, were used to formulate the DPPs. For parenteral administration, cKK‐E10 based DPPs containing hOTC and hEPO mRNAs were evaluated by intravenous and intramuscular routes, respectively. Since DPPs are not directly suitable for parenteral dosing, only RDPPs were administered for these routes. For mucosal delivery, however, both DPPs (administered as‐is) and RDPPs were feasible. Accordingly, both dosage form presentations were assessed using FFLuc mRNA‐based products formulated with ionizable lipids cKK‐E10 and GA‐91 for pulmonary and sublingual routes, respectively.

Parenteral routes demonstrated preserved therapeutic efficacy post spray drying, with in vivo mRNA expression profiles comparable to liquid formulations. Following intravenous and intramuscular administration, the RDPP produced hOTC protein levels in the liver (Figure 6a) and hEPO protein levels in the serum (Figure 6d), respectively that were comparable to those achieved with the corresponding LC products prior to spray drying. Importantly, spray drying maintained both the local hepatic expression of hOTC, a non‐secretory protein that remains confined to the liver, and the systemic secretion of hEPO into circulation, demonstrating that the process preserved the distinct biological fates of these two model proteins. Most critically, following intravenous administration, the preserved hOTC expression in the liver confirms that spray drying did not disrupt serum protein binding or the subsequent biodistribution and uptake of reconstituted LNPs to hepatic tissue, critical processes that govern apolipoprotein E (ApoE) binding mediated hepatic tropism and low‐density lipoprotein receptor (LDLR) dependent uptake following systemic injection. This was further validated by quartz crystal microbalance with dissipation monitoring (QCM‐D) evaluation, which directly measured the binding between ApoE and LNPs. Although RDPP exhibited a slightly higher negative frequency shift (Figure 6b) and marginally increased binding efficiency (Figure 6c) compared to LC, the comparable hOTC expression levels between RDPP and LC confirmed that these minor differences in binding kinetics did not translate to functionally significant changes in hepatic delivery or protein expression, demonstrating that the spray drying process did not meaningfully disrupt serum protein interactions. These results collectively confirm that the spray drying process did not compromise LNP structure, mRNA integrity, or the overall biodistribution and efficiency of intracellular delivery and post translational processing, factors that are typically vulnerable to shear forces, dehydration stress, and thermal exposure during particle engineering. The preservation of both tissue localized (hOTC) and secretory (hEPO) protein expression following intravenous and intramuscular administration, respectively supports the robustness of spray drying as a manufacturing process for DPP development and underscores its suitability for creating stable formulations compatible with potential standard refrigeration storage temperatures without diminishing therapeutic potency.

**FIGURE 6:**
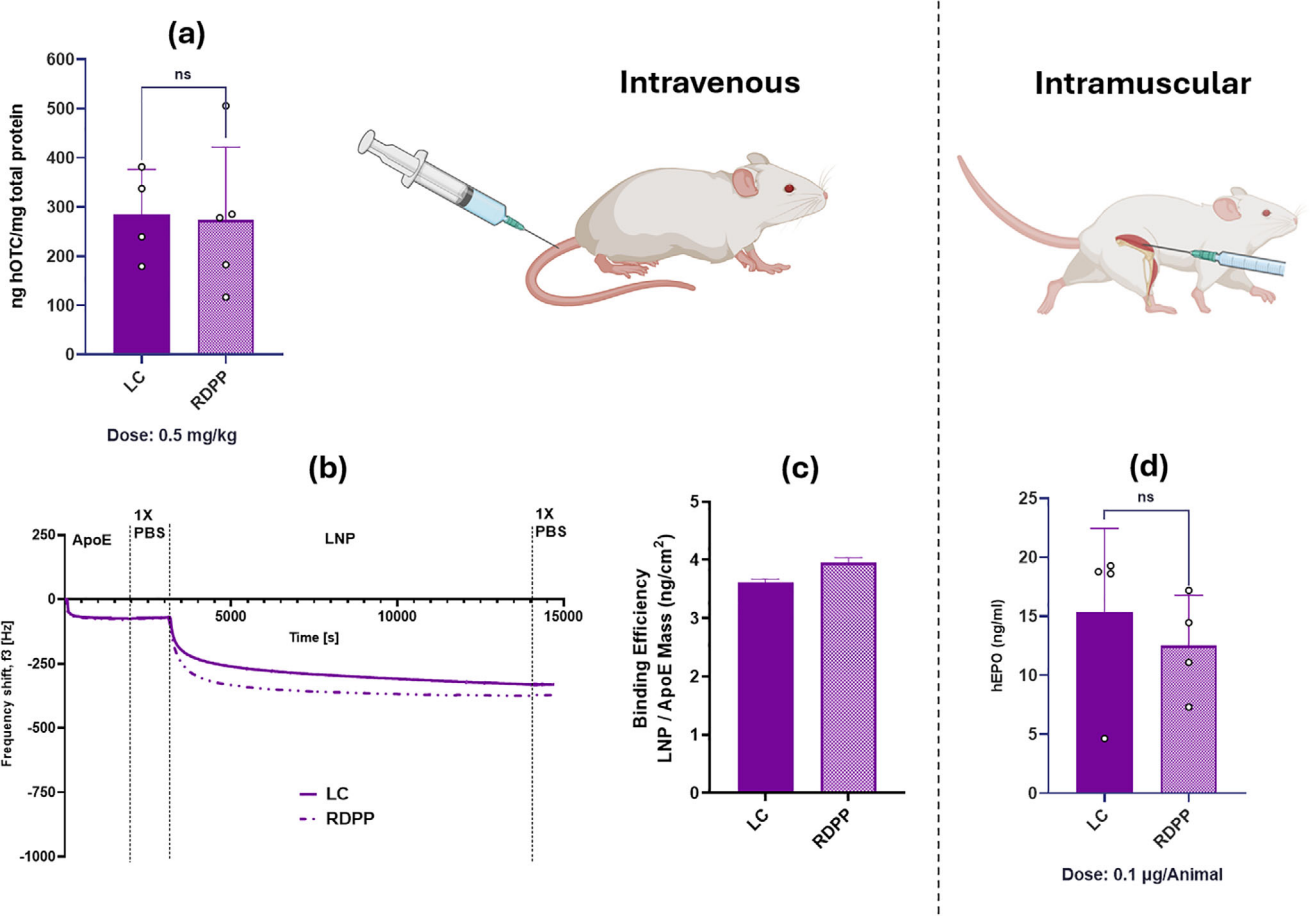
In vivo therapeutic efficacy and serum protein binding of mRNA‐LNP RDPPs via parenteral routes. (a) Hepatic hOTC protein expression following intravenous administration (0.5 mg/kg) showed comparable levels between LC and RDPP. (b) QCM‐D frequency shift and (c) binding efficiency measurements demonstrated preserved ApoE‐LNP interactions post‐spray drying. (d) Serum hEPO protein levels following intramuscular injection (0.1 µg/animal) remained equivalent between LC and RDPP. No hOTC or hEPO protein was detected in saline‐treated animals. Results confirmed that spray drying preserved LNP structure, mRNA integrity, biodistribution, and therapeutic potency for both tissue‐localized and secretory protein expression. Student's T‐Test with Welch's Correction (n = 4). ns denotes statistical insignificance.

Mucosal delivery routes revealed transformative potential for thermostable therapeutics. The spray drying process demonstrated exceptional preservation of protein expression for mucosal delivery across pulmonary and sublingual routes. Most significantly for clinical translation, the successful performance of the DPP as‐is for pulmonary administration represents a paradigm shift for thermostable product development, eliminating the need for cold chain storage and reconstitution steps that are typically required for traditional liquid formulations. Following intra‐tracheal administration, both RDPP and LC products exhibited comparable levels of FFLuc expression predominantly localized to the lungs, confirming that the spray drying process followed by reconstitution maintained the structural and functional integrity of the LNPs (Figure 7a,b). The background images of saline treated animals didn't show any detectable bioluminescence in any of the organs (Figure S5a). Also, enzyme levels across all treatment groups were not significantly different from saline‐treated controls, indicating no evidence of hepatotoxicity associated with any of the formulations tested (Figure S5b). The preservation of LNP morphology and encapsulation efficiency during spray drying is particularly noteworthy, as it demonstrates that critical quality attributes necessary for effective pulmonary delivery, including particle size distribution, surface properties, and payload stability, remain intact throughout the dehydration and particle formation processes followed by rehydration during reconstitution of the DPP. The DPP administered as‐is without reconstitution demonstrated well distributed FFLuc expression throughout the lungs, validating the concept of direct DPP inhalation delivery with profound implications for global therapeutic access (Figure 7a,b). This confirms that spray dried formulations can bypass typical limitations associated with liquid aerosol generation, including LNP aggregation during nebulization, thermal degradation during storage, and the complexity of maintaining cold chain logistics in resource limited settings. The lung specific biodistribution observed via pulmonary administration, with no detectable FFLuc bioluminescence in other organs, demonstrates excellent lung specificity and suggests a favorable safety profile due to absent off target delivery. These findings demonstrate the feasibility of direct pulmonary DPP administration and warrant future studies evaluating aerosol performance using pharmaceutically relevant inhalation devices.

**FIGURE 7:**
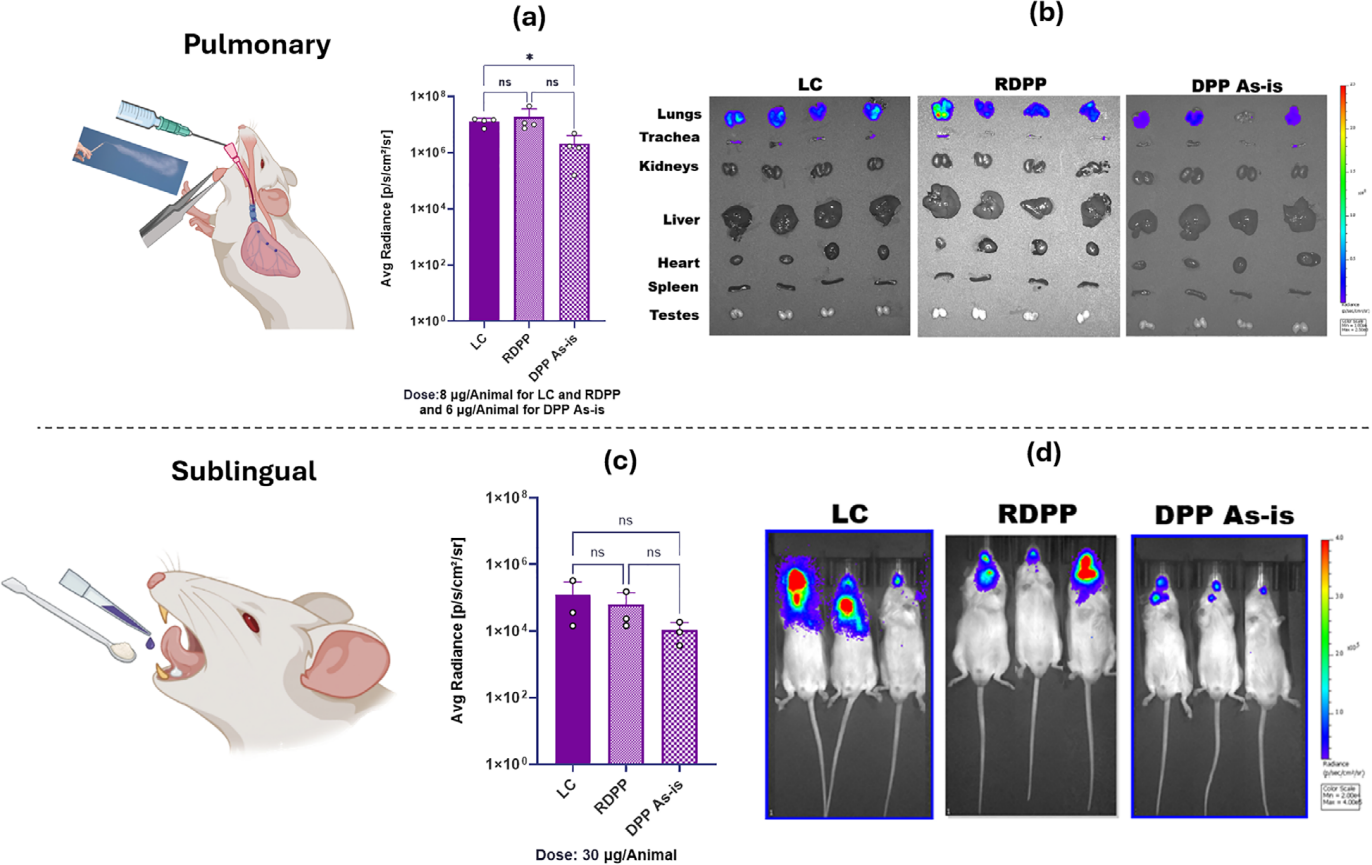
Mucosal delivery of mRNA‐LNP RDPPs or DPPs via pulmonary and sublingual routes. (a) FFLuc expression quantification and (b) biodistribution imaging following intratracheal administration (6‐8 µg dose) demonstrated comparable lung‐specific expression between LC, RDPP, and DPP administered as‐is, with no off‐target delivery to other organs. (c) FFLuc expression quantification and (d) whole‐body bioluminescence imaging following sublingual administration (30 µg dose) showed robust expression with RDPP comparable to LC, while DPP as‐is exhibited reduced but detectable expression. No bioluminescence signal was detected in saline‐treated animals. Pulmonary delivery of DPP as‐is represents a paradigm shift for thermostable therapeutics, eliminating cold chain requirements and reconstitution steps while maintaining therapeutic efficacy. Student's T‐Test with Welch's Correction (*n* = 4 and 3 for pulmonary and sublingual, respectively). Asterisks denote p‐values: ^*^ *p* < 0.05. ns denotes statistical insignificance.

Sublingual administration presented significant translational considerations for DPPs, with route specific variability inherent to the sublingual delivery pathway observed across all tested products. Notably, the RDPP retained robust protein expression comparable to that achieved with the LC product, demonstrating that spray drying process and subsequent reconstitution of the generated DPP does not compromise the LNP integrity for this alternative route (Figure 7c,d). The background image of saline treated animal didn't show any detectable bioluminescence (Figure S6). While the DPP administered as‐is via the sublingual route showed lower expression compared to both LC and RDPP formulations, the demonstration of detectable protein expression from the thermostable DPP without reconstitution is noteworthy. Also, it is critical to acknowledge significant limitations associated with administering dry powder as‐is via the sublingual route in small animal species such as mice. The anatomical constraints of the murine oral cavity, combined with the limited saliva volume and rapid clearance mechanisms inherent to small rodent models, present substantial challenges for effective dry powder retention and dissolution at the sublingual mucosa. The inherent difficulty in precisely depositing and maintaining dry powder particles in the sublingual space of mice, coupled with the rapid mucociliary clearance and swallowing reflexes characteristic of small animal physiology, likely contributed to the reduced expression observed with the DPP formulation. These limitations are particularly pronounced in small animal models and may not accurately reflect the performance potential in larger animal species or human subjects, where greater oral cavity volume, increased saliva production, and different mucosal physiology could substantially enhance dry powder retention and therapeutic efficacy. Despite these preclinical limitations, the demonstration of detectable protein expression from the DPP opens unprecedented opportunities for developing patient compliant thermostable mRNA‐LNP products that do not require cold chain maintenance or reconstitution procedures, fundamentally transforming the accessibility of advanced biopharmaceuticals across diverse clinical and geographic settings. Collectively, the successful demonstration of DPP efficacy as‐is across both pulmonary and sublingual routes establishes this technology as a cornerstone for next generation thermostable biopharmaceutical products that combine formulation convenience with therapeutic potency, with particular promise for pulmonary delivery applications and potential for sublingual administration in larger animal models and clinical settings.

### 2.5 Exceptional Long‐Term Stability Under Pharmacy‐Friendly Storage Conditions Establishes DPP as Transformative Platform Technology

A comprehensive long‐term stability study was designed to systematically evaluate the enhancement in mRNA‐LNP stability achieved using DPP when stored under refrigerated conditions (2°C–8°C) for 3–3.5 years. The study encompassed a diverse range of mRNA constructs and examined both non‐biodegradable and biodegradable ionizable lipid‐based formulations to provide a thorough assessment across different molecular architectures. Lead DPP formulations were prepared to achieve a post‐reconstitution trehalose concentration of 5% w/v, utilizing LNPs encapsulating various mRNA constructs, including hEPO, monovalent influenza (Flu), quadrivalent Flu, and respiratory syncytial virus (RSV). To ensure broad applicability of the findings, two chemically distinct ionizable lipid systems were employed: cKK‐E10 (non‐biodegradable) and GA‐91 (biodegradable), enabling comparative assessment of DPP stability characteristics across different lipid chemistries. Importantly, assessing the ability of DPP to maintain stability of both the mRNA and the degradable ionizable lipid is crucial, as lipid degradation during long‐term storage can compromise LNP structural integrity and transfection efficiency, ultimately affecting therapeutic efficacy. All quality attributes, including colloidal stability and structural morphology of the mRNA‐LNPs, chemical stability of the mRNA and ionizable lipid, and protein expression capability, were preserved across various mRNA constructs and ionizable lipid‐based DPPs over the stability period.

The colloidal stability parameters, including particle size, encapsulation efficiency, and PdI, were maintained for up to 3.5 years for the RDPP of the cKK‐E10‐based DPP formulated with hEPO mRNA (Figure 8a). Similarly, the chemical stability of the mRNA, as assessed by capillary electrophoresis (CE) analysis, was preserved throughout the stability period. Furthermore, protein expression data correlated well with the retained mRNA integrity, with no significant decline observed over the stability period (Figure 8b). Comparable stability profiles were observed for viral antigen expressing mRNA constructs – monovalent Flu (Figure 8c) and RSV (Figure 8d) – where both colloidal stability of the mRNA‐LNPs and chemical stability of the mRNA were maintained over 3.5 years at 2°C–8°C storage. The morphological characteristics of the mRNA‐LNPs were also preserved for this representative monovalent Flu‐based product throughout the stability period, with both the LC and RDPP stability samples showing their characteristic multi‐lamellar and core‐based structures, as confirmed by cryo‐transmission electron microscopy (Cryo‐TEM) analysis (Figure 9). To overcome the limitations of CE analysis in resolving intact mRNA stability, the RSV mRNA‐based RDPP was additionally evaluated using reversed‐phase ion‐pair ultra‐performance liquid chromatography (RP‐IP‐UPLC) to differentiate between mRNA fragment formation and mRNA‐lipid adduct species [47]. The intact mRNA was well preserved, with no significant increase in mRNA fragments or mRNA‐lipid adducts detected over the stability period (Figure 8d).

**FIGURE 8:**
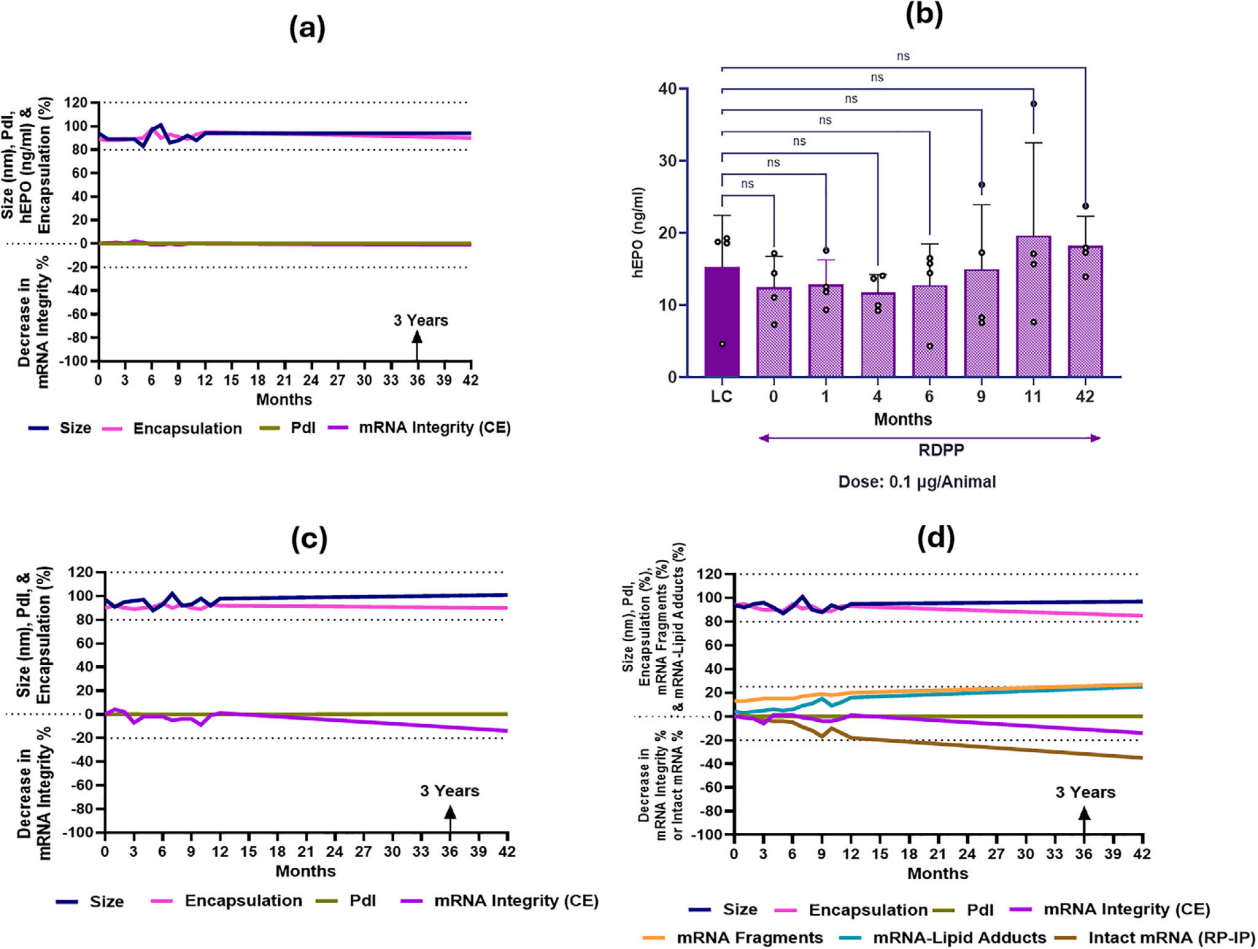
Long‐term stability of mRNA‐LNP DPPs containing non‐biodegradable ionizable lipid cKK‐E10 stored at 2°C–8°C for up to 3.5 years. DPPs formulated with various mRNA constructs maintained all critical quality attributes throughout the storage period. RDPPs were analyzed for colloidal stability parameters (particle size, PdI, encapsulation efficiency), mRNA chemical integrity by CE, and functional protein expression. (a) hEPO mRNA‐LNP DPP colloidal and chemical stability parameters and (b) corresponding serum hEPO protein expression following intramuscular administration. No hEPO protein was detected in saline‐treated animals. Statistical analysis by Student's T‐Test with Welch's Correction (*n* = 4); ns denotes no statistical significance. (c) Monovalent influenza mRNA DPP stability profile. (d) RSV mRNA‐LNP DPP stability with RP‐IP‐UPLC analysis confirming preservation of intact mRNA and absence of mRNA fragments or lipid adducts.

**FIGURE 9:**
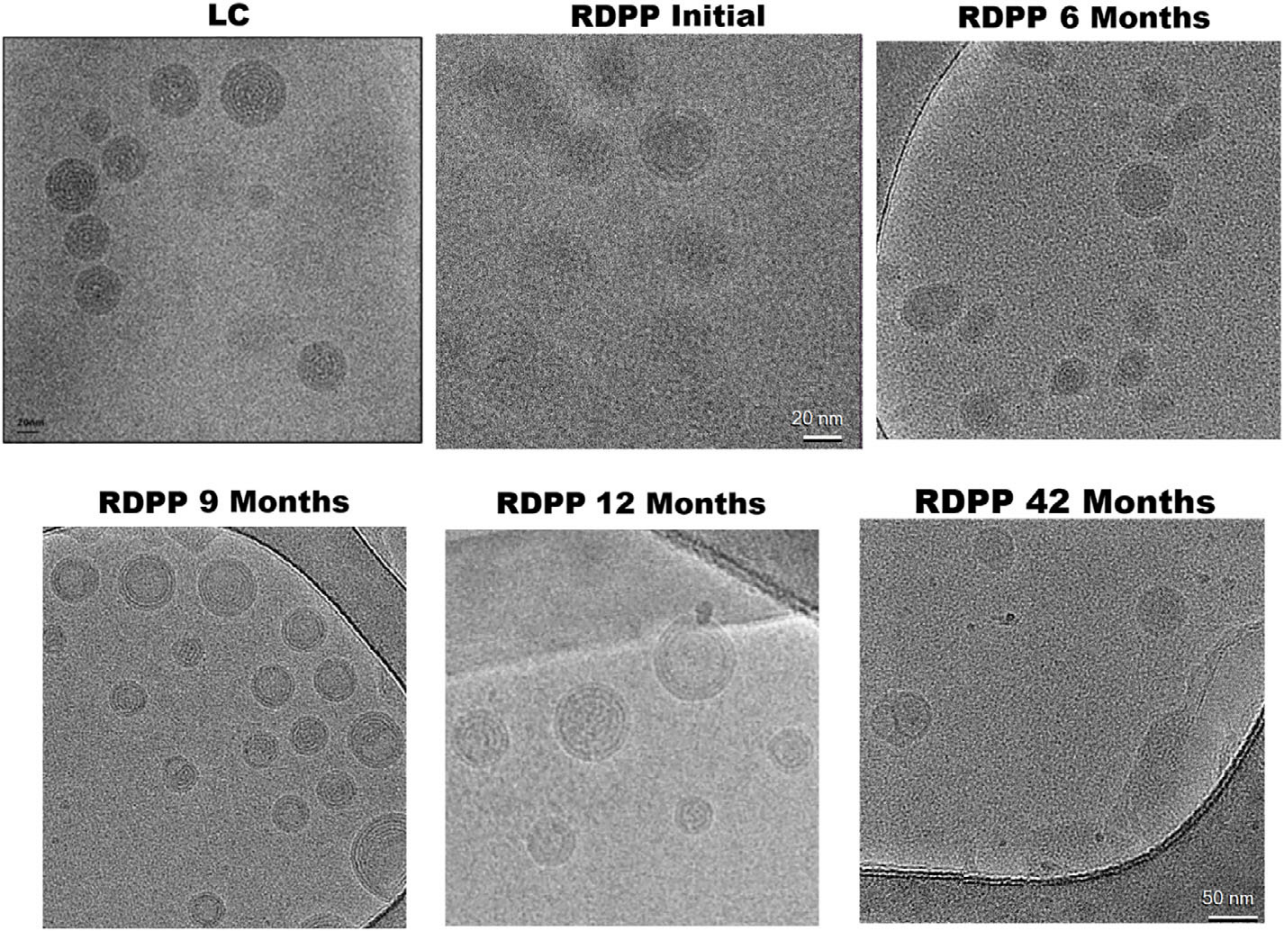
Cryo‐TEM analysis of mRNA‐LNP morphology during long‐term stability. Cryo‐TEM images of LC and RDPP stability samples of cKK‐E10‐based monovalent Flu mRNA‐LNP formulations at representative time points over 3.5 years at 2°C–8°C storage. Both LC and RDPP samples retained their characteristic multi‐lamellar and core‐based LNP structures throughout the stability period, confirming that spray drying and long‐term refrigerated storage did not compromise the structural morphology of the mRNA‐LNPs.

The stability of the biodegradable ionizable lipid‐based DPP was further evaluated using the GA‐91‐based product formulated with quadrivalent Flu mRNAs. As shown in Figure 10a, the colloidal stability parameters, including particle size, encapsulation efficiency, and PdI, were maintained for up to 3 years for the RDPP of the GA‐91‐based DPP. Similarly, the chemical stability of the mRNA, as assessed by CE analysis, was preserved throughout the stability period. To further characterize mRNA integrity, RP‐IP‐UPLC analysis confirmed the absence of any significant increase in mRNA fragments or mRNA‐lipid adduct species over the stability period (Figure 10b). Importantly, the chemical stability of the GA‐91 ionizable lipid itself was also maintained, as evidenced by UPLC‐charged aerosol detector (UPLC‐CAD) analysis, with no significant degradation detected over 3 years (Figure 10c). In contrast, the LC formulation exhibited a significant decline in mRNA integrity post 6 months, as measured by CE analysis (Figure 10a). RP‐IP‐UPLC analysis further revealed a significant increase in mRNA fragments and a corresponding decline in intact mRNA for the LC, although mRNA‐lipid adducts remained stable (Figure 10b). Moreover, the degradation of the GA‐91 ionizable lipid was significantly accelerated in the LC formulation, with more than 90% degradation observed within just 9 months (Figure 10c). Collectively, these results demonstrate the superior ability of DPP to preserve both mRNA and ionizable lipid stability across biodegradable lipid‐based formulations over extended storage periods compared to conventional liquid formulations.

**FIGURE 10:**
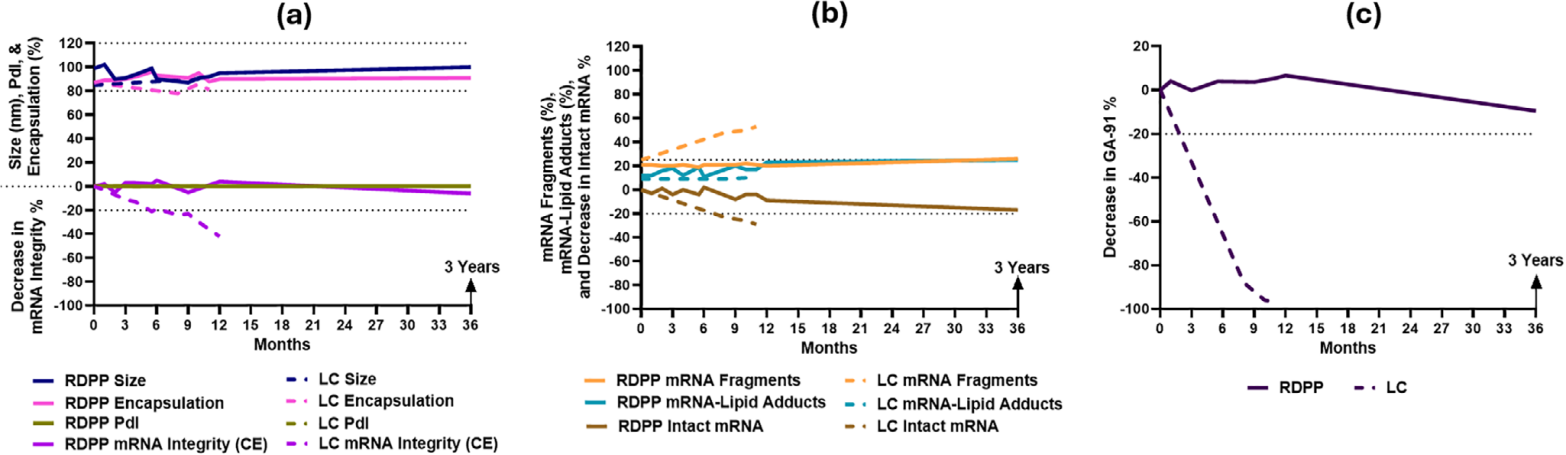
Comprehensive long‐term stability comparison of mRNA‐LNP DPPs versus LC formulation containing biodegradable ionizable lipid GA‐91 stored at 2°C–8°C. (a) Quadrivalent Flu mRNA‐LNP DPPs maintained colloidal stability and mRNA integrity over 3 years upon reconstitution, while corresponding LC formulation showed significant mRNA degradation after 6 months. (b) RP‐IP‐UPLC analysis demonstrating increased mRNA fragmentation in LC with intact mRNA preserved in DPP formulations. (c) UPLC‐CAD analysis showing preservation of GA‐91 ionizable lipid integrity in DPPs versus >90% lipid degradation in LC within 9 months. These results establish DPP as a superior platform for long‐term stabilization of mRNA‐LNPs containing biodegradable ionizable lipids under pharmacy‐compatible refrigerated storage conditions.

These comprehensive stability data demonstrate that DPP technology successfully addresses the critical challenge of long‐term storage stability for mRNA‐LNP vaccines and therapeutics under standard refrigerated conditions. The preservation of all critical quality attributes including colloidal stability, structural morphology, mRNA integrity, protein expression capability, and ionizable lipid stability across diverse mRNA constructs and both biodegradable and non‐biodegradable ionizable lipid systems for up to 3.5 years represents a significant advancement over conventional liquid formulations. The dramatic contrast observed with the liquid product, where substantial mRNA degradation and greater than 90% ionizable lipid degradation occurred within 6 to 9 months, underscores the transformative potential of DPP technology. These findings have profound implications for the clinical and commercial viability of mRNA vaccines and therapeutics, potentially enabling simplified cold chain logistics, reduced manufacturing frequency, extended product shelf life, and improved global accessibility of mRNA‐based therapeutics. Furthermore, the demonstrated stability of biodegradable ionizable lipids within DPP formulations addresses a key limitation that has historically constrained the development of next generation, biocompatible LNP delivery systems. Collectively, these results position DPP as an enabling platform technology that could accelerate the translation of mRNA medicine from laboratory to patient, particularly in resource limited settings where cold chain infrastructure remains challenging.

## 3 Conclusions

This study establishes spray drying as a robust and scalable manufacturing platform for thermostable mRNA‐LNP solid dosage form, addressing critical barriers to the global deployment of mRNA vaccines and therapeutics including cold chain dependency and limited patient‐compliant administration options. Through systematic excipient screening, trehalose was identified as the optimal sugar, forming a stable amorphous matrix that entraps mRNA‐LNPs within the DPP core, preserving structural integrity, encapsulation efficiency, and biological activity through dehydration and rehydration cycles. The DPP platform demonstrated remarkable versatility, supporting both parenteral delivery following reconstitution and non‐parenteral delivery via reconstitution or direct DPP administration, while maintaining in vivo protein expression profiles comparable to LCs. Notably, direct DPP administration via pulmonary and sublingual routes represents a paradigm shift, providing patient‐compliant thermostable alternatives that eliminate cold chain requirements for non‐parenteral delivery. Long‐term stability data over 3 to 3.5 years at 2°C–8°C across diverse mRNA constructs and both biodegradable and non‐biodegradable ionizable lipid systems confirm the broad applicability and superiority of the platform over conventional liquid formulations. The enhanced stabilization of biodegradable ionizable lipids within the DPP further expands the design space for next generation LNP systems. Collectively, DPP technology positions spray drying as a cornerstone manufacturing approach that can accelerate the translation of mRNA vaccines and therapeutics from laboratory to patients, particularly enabling thermostable, patient‐compliant delivery options for non‐parenteral routes, fundamentally transforming the accessibility, shelf life, and clinical utility of this transformative therapeutic modality.

## 4 Experimental Section

### 4.1 Materials

FFLuc, hOTC, hEPO, Monovalent Flu (Tasmania strain), and Quadrivalent Flu (Tasmania, Phuket, Wisconsin, and Washington strains) mRNAs; and ionizable lipids – cKK‐E10 [48] and GA‐91 [49, 50, 51] were synthesized internally at Sanofi. The structures of ionizable lipids are shown in Figure S1. 1,2‐Dimyristoyl‐rac‐glycero‐3‐methoxypolyethylene glycol‐2000 (DMG‐PEG‐2000) and 1,2‐dioleoyl‐sn‐glycero‐3‐phosphoethanolamine (DOPE) were obtained from NOF America Corporation (White Plains, NY, USA). Cholesterol was sourced from Millipore Sigma (Burlington, MA, USA). Ethanol and sugars – Lactose, xylitol, mannitol, sucrose, and trehalose were acquired from Fisher Scientific. Citrate buffer (pH 4.5) and sodium chloride solution were purchased from Boston Scientific (Marlborough, MA, USA).

### 4.2 Preparation of DPPs and RDPPs

mRNA‐LNPs were prepared as described previously [52], by rapid T‐mixing of an ethanolic lipid mixture comprising ionizable lipid, DOPE, cholesterol, and DMG‐PEG‐2000 at a molar ratio of 40:30:28.5:1.5 with an aqueous mRNA solution in citrate buffer pH 4.5 using gear pumps, at a fixed ionizable lipid‐to‐nucleotide molar ratio of 4:1 to produce uniform LNP dispersions. The LNP dispersion was subsequently processed by ultrafiltration and diafiltration into a 20% v/v ethanol containing sugars at concentrations yielding 1.25–7% w/v upon reconstitution. The diafiltered suspension was spray dried at 0.3 mg/mL mRNA concentration using a Büchi B‐290 spray dryer with process parameters as summarized in Table 1 yielding DPPs in the range of 0.3–1.2% w/w mRNA loading. The resulting DPPs were reconstituted with water for injection to 0.2 mg/mL mRNA concentration to generate RDPPs.

**TABLE 1:** Spray drying conditions.

Parameter	Open Loop	Closed Loop (Inert)
Nozzle Diameter (mm)	0.7	0.7
Inlet Temperature (°C)	65	85
Aspirator (%)	100	100
Pump (%)	25	25
Outlet Temperature (°C)	37–40	37–40
Pressure (mbar)	−30 to −50	−30 to −50
Drying Gas	Air	Nitrogen
Atomization Gas	Nitrogen	Nitrogen

### 4.3 Characterization of LCs, DPPs, and RDPPs

DPP particle size was measured by laser diffraction (Mastersizer 3000, Malvern Panalytical). Moisture content of DPP was determined by LOD (45°C, 48 h, vacuum desiccator) and KF titration. Hydrodynamic diameter, PdI, and zeta potential of mRNA‐LNPs in LCs and RDPPs were determined by dynamic light scattering (DLS) (Zetasizer Nano ZS, Malvern Panalytical). The zeta potential was in the range of −6–−10 mV. Zeta potential was intermittently monitored throughout the storage period and did not show any consistent or progressive change before versus after storage, indicating that the surface charge properties of the particles were preserved following spray drying, reconstitution, and long‐term storage. RDPPs were also examined by light microscopy at 400× magnification to identify visible aggregates. mRNA concentration and encapsulation efficiency of RDPPs were quantified using the RiboGreen assay (Thermo Fisher Scientific) following the manufacturer's protocol. mRNA integrity was assessed by CE analysis (Agilent), and intact mRNA, mRNA fragments, and mRNA‐lipid adducts were measured by RP‐IP‐UPLC‐Ultra violet (UV) (Waters Acquity) using a DNAPac RP column (2.1 × 100 mm, 4 µm, Thermo Scientific Fisher Ref. 088923). Lipid concentration was determined by UPLC‐CAD (Waters Acquity) using an Infinity Lab Poroshell 120 Phenyl‐Hexyl column (2.1 × 50 mm, 1.9 µm, Agilent P/N 699675–9).

### 4.4 Morphological Evaluation of LCs, DPPs, and RDPPs

DPP surface and shape morphology was examined by SEM using a JEOL JCM‐6000 Benchtop instrument. Approximately 1–2 mg of sample was deposited onto a sample stage affixed with SEM imaging tape and coated with a thin layer of gold using a sputter coater. Images were acquired in high vacuum mode using secondary electron imaging (SEI) at an accelerating voltage of 10 kV with standard filament and probe currents. Samples were imaged at magnifications of 30×, 60×, 500×, and 1000×. LCs and RDPPs were examined by Cryo‐TEM imaging. Briefly, samples were prepared by plunge‐freezing vitrification and imaged using brightfield Cryo‐TEM techniques. Samples were applied to grids, blotted, and rapidly plunged into liquid ethane to vitrify. Each LNP formulation was vitrified on mesh grids with and without a continuous carbon film to optimize visualization conditions. Imaging was conducted on a transmission electron microscope. Micrographs were acquired at nominal magnifications of 49,000× and 130,000×.

### 4.5 QCM‐D Evaluation of LCs and RDPPs

Measurements were performed using Au‐coated sensors (QSX301, Biolin Scientific) with a 4.95 MHz resonance frequency. Sensor preparation involved sequential cleaning steps: 10 min UV‐Ozone treatment, 5 min base piranha treatment, thorough rinsing with MilliQ water, N2 drying, and a final 10 min UV‐Ozone treatment. An E4 QCM‐D instrument (Q‐Sense Inc., Gothenburg, Sweden) was used to monitor changes in resonance frequency (ΔF) and dissipation (ΔD) for odd overtones, with all measurements referenced to PBS buffer‐equilibrated baseline values. Solution delivery was controlled using a peristaltic pump operating at 40 µL/min. ApoE adsorption was initiated by introducing 0.1 mg/mL ApoE in PBS over the sensors [53]. Following 20 min of continuous flow, an intermittent pumping protocol was implemented (50 µL continuous flow followed by 4–9 min pause) to conserve material. After 80 min of adsorption, the ApoE layers were equilibrated with PBS buffer (40 µL/min, ∼20 min). LNP‐ApoE interactions were investigated by introducing 0.05 mg/mL LNP in PBS over the equilibrated ApoE layer. The flow protocol consisted of 10 min continuous pumping followed by intermittent delivery (50 µL over 1 min, followed by 4‐min pause) for 80 min total interaction time. The system was then rinsed with PBS buffer under continuous flow conditions.

### 4.6 Solid State Characterization of DPPs

PXRD was performed using powder X‐ray diffractometer (Rigaku MiniFlex 600) at 40 kV, 15 mA with SC‐70 detector to determine the crystalline or amorphous nature of the DPPs. The X‐ray pattern was collected in the angular range of 3< 2θ < 90° in the step scan mode (step width 0.02°, scan rate 10°/per minute). The thermal behavior of DPP samples was analyzed using MDSC using TA Instruments DSC Q2000 attached with refrigerated cooling system (RCS90). Following initial equilibration at 35.00°C and a 5.00 min isothermal period under modulation conditions of ±1.00°C every 60 s, data collection was initiated. The experimental profile consisted of three distinct segments – an initial dynamic heating ramp to 200.00°C (Cycle 1), a cooling ramp back to 35.00°C (Cycle 2), and a final reheating ramp to 200.00°C (Cycle 3). All temperature ramps were performed at a rate of 2.00°C/min, separated by 5.00 min isothermal holds to stabilize the thermal history.

### 4.7 Animal Studies

Two different mouse strains were utilized – CD‐1 and BalbC (Charles River Laboratories) for intravenous/mucosal and intramuscular routes of administration, respectively. Animals were maintained in pathogen‐free conditions with a 12‐h light/dark cycle and provided ad libitum access to food and water. LCs and RDPPs were administered as single doses intravenously at 1 mg RNA /kg in a 5 mL/kg injection volume via tail vein or intramuscularly at 0.1 µg mRNA per animal in a 30 µL injection volume into the quadriceps muscle. Animals were euthanized 24 and 6 h post‐dose for intravenous and intramuscular administration, respectively using carbon dioxide asphyxiation followed by thoracotomy. Whole liver was harvested, with 3–6 liver biopsies (4 or 8 mm punches) collected per animal for intravenous route, whereas whole blood (∼50 µL) was collected by tail snip or saphenous vein for intramuscular route. Liver tissue and serum samples were stored at −70°C for subsequent hOTC and hEPO protein expression analysis, respectively by ELISA. LC or RDPP (8 µg mRNA/Animal) and DPP (6 µg mRNA/Animal) were administered intratracheally using catheter and dry powder insufflators, respectively for the pulmonary route. The intratracheal dose of mRNA in DPP was slightly lower as compared to that in LC and RDPP due to dosing constraints in mice. LC, RDPP, and DPP were administered at 30 µg mRNA /Animal dose for sublingual route. After 24 h of intratracheal or sublingual administration, all animals were dosed with 3 mg of D‐Luciferin /animal via subcutaneous (SC) injection. Post euthanasia, whole body imaging was conducted for sublingual route, whereas lungs, trachea, kidneys, heart, spleen, tests were harvested and imaged in a petri dish using IVIS imaging for pulmonary route.

### 4.8 Ethics Statement

All animal studies were conducted at Alpha Preclinical (North Grafton, MA) in accordance with US National Institutes of Health guidelines and relevant local, state, and federal regulations. Experimental protocols were approved by Tufts University's Institutional Animal Care and Use Committee (IACUC) and adhered to ethical guidelines for animal research.

### 4.9 Statistical Analysis

GraphPad PRISM was used to perform statistical analysis and plot the graphs. Comparisons between the means (± SD) shown in the bar plots were performed using either Student's T‐Test with Welch's correction.

## Author Contributions

The manuscript was written through contributions of all authors. All authors have given approval to the final version of the manuscript. **Ashish Sarode**: Original Conceptualization, Methodology, Investigation, Supervision, Validation, Writing – original draft, review & editing, and visualization. **Priyal Patel**: Investigation, Experimental. **Shrirang Karve**: Supervision, Draft review & editing, and Project administration. **Frank DeRosa**: Project administration, Draft review & editing. **Regis Gervier**: Project administration, Draft review & editing.

## Funding

The research included in the manuscript was funded by Sanofi.

## Conflicts of Interest

The authors declare no conflicts of interest.

## Supporting information




**Supporting File**: advs78751‐sup‐0001‐SuppMat.docx.

## Data Availability

The data that support the findings of this study are available from the corresponding author upon reasonable request.
