# Supplementary material for: Spray‐Dried mRNA‐LNP Powder Enabling Multi‐Route Delivery With 3‐Year Refrigerated Stability
Source: Adv Sci (Weinh). 2026 Nov 10:e78751. Online ahead of print. doi: 10.1002/advs.78751 (PMC13750010; doi:10.1002/advs.78751)
Supplement: Supplementary file 1 [file ADVS-9999-e78751-s001.docx]

**Table S1. Comparison of ethanol treatment methods for evaluating mRNA-LNP entrapment within DPP matrix.**

| **Step** | **Vortexed in ethanol** | **Overnight Stirring** |
| --- | --- | --- |
| 1 | Add 5 mL ethanol to 500 mg dry powder in a 50 mL tube. Vortex at high speed for 30 seconds. | Add 20 mL ethanol to 500 mg dry powder in a small flat-bottom glass bottle with a small stir bar. |
| 2 | Centrifuge for 1 min at 3600 RPM. Collect supernatant in another 50 mL tube. | Cover glass bottle with aluminum foil and stir overnight at full speed on a stir plate. |
| 3 | Add 5 mL ethanol to the remaining pellet and vortex for 30 seconds. Repeat steps 2 and 3 three more times. | Transfer solution to a 50 mL centrifuge tube and centrifuge for 1 min at 3600 RPM. Discard supernatant. |
| 4 | Dry pellet in vacuum desiccator at RT for 2 days until completely dried. | Dry pellet in vacuum desiccator at RT for 2 days until completely dried. |
| **Total Ethanol Volume** | 20 mL | 20 mL |
| **Treatment Duration** | ~2 minutes (4 × 30 sec vortexing) | Overnight (~12–16 hours) |
| **Mechanism** | Vigorous mechanical agitation with brief ethanol exposure | Gentle continuous stirring with prolonged ethanol exposure |

**Fig. S1. Chemical structures of ionizable lipids.** (A) cKK-E10 and (B) GA-91.

**Fig. S2. SEM images of DPP samples**

mRNA-LNP DPP at 7% w/v post-reconstitution mannitol concentration

mRNA-LNP DPP at 2.5% w/v post-reconstitution mannitol concentration

mRNA-LNP DPP at 7% w/v post-reconstitution sucrose concentration

mRNA-LNP DPP at 2.5% w/v post-reconstitution sucrose concentration

mRNA-LNP DPP at 7% w/v post-reconstitution trehalose concentration

mRNA-LNP DPP at 2.5% w/v post-reconstitution trehalose concentration

mRNA-LNP DPP at 1.25% w/v post-reconstitution trehalose concentration

DPP of mannitol only

DPP of sucrose only

DPP of Trehalose only

**Fig. S3.** PXRD plots of DPP samples. Note: four large peaks showing beyond ~ 35 deg are coming from the OHC4 enclosure stage (blank).

mRNA-LNP DPP at 7% w/v post-reconstitution mannitol concentration

mRNA-LNP DPP at 2.5% w/v post-reconstitution mannitol concentration

mRNA-LNP DPP at 7% w/v post-reconstitution sucrose concentration

mRNA-LNP DPP at 2.5% w/v post-reconstitution sucrose concentration

mRNA-LNP DPP at 7% w/v post-reconstitution trehalose concentration

mRNA-LNP DPP at 2.5% w/v post-reconstitution trehalose concentration

mRNA-LNP DPP at 1.25% w/v post-reconstitution trehalose concentration

DPP of mannitol only

DPP of sucrose only

DPP of trehalose only

**Fig. S4.** MDSC profiles of DPPs

mRNA-LNP DPP at 7% w/v post-reconstitution mannitol concentration

mRNA-LNP DPP at 2.5% w/v post-reconstitution mannitol concentration

mRNA-LNP DPP at 7% w/v post-reconstitution sucrose concentration

mRNA-LNP DPP at 2.5% w/v post-reconstitution sucrose concentration

mRNA-LNP DPP at 7% w/v post-reconstitution trehalose concentration

mRNA-LNP DPP at 2.5% w/v post-reconstitution trehalose concentration

mRNA-LNP DPP at 1.25% w/v post-reconstitution trehalose concentration

**Fig. S5.** (a) Background bioluminescence images of saline treated animals using intratracheal administration. (b) Liver function enzymes ALT, AST, and ALP levels following intratracheal administration (6-8 μg dose) of LC, RDPP, DPP administered as-is, and saline control.

(a)

(b)

**Fig. S6.** Background bioluminescence image of saline treated animal using sublingual administration.
